# A technical modification for percutaneous tracheostomy: prospective case series study on one hundred patients

**DOI:** 10.1186/1749-7922-6-35

**Published:** 2011-11-02

**Authors:** Joao B Rezende-Neto, Argenil J Oliveira, Mario P Neto, Fernando A Botoni, Sandro B Rizoli

**Affiliations:** 1 Universidade Federal de Minas Gerais and Risoleta Tolentino Neves Hospital, Brazil; 2 Risoleta Tolentino Neves Hospital, Brazil; 3 University of Toronto and Sunnybrook Hospital, Canada

**Keywords:** Percutaneous tracheostomy, technique.

## Abstract

The purpose of this study is to describe a technical modification of percutaneous tracheostomy that combines principles of the Percu Twist™ and the Griggs-Portex^® ^methods in a reusable kit. One hundred patients underwent the procedure. There were no false passage, tube misplacement, or deaths related to the procedure. There were two minor bleedings managed conservatively. The technical modification described in this study is safe and simple to execute.

## Introduction

Tracheostomy is one of the most frequently performed surgical procedures in intensive care unit (ICU) patients [1]. Percutaneous tracheostomy has gained widespread acceptance as an alternative to open surgical tracheostomy with the advantage of "bedside" performance and minimal morbidity [2-4]. Most percutaneous tracheostomy methods incorporate the Seldinger technique to gain initial access to the tracheal lumen. However, after that initial step, a number of variations have been described [2,4-10]. The method introduced by Ciaglia and colleagues in 1985, has become the most popular technique for percutaneous tracheostomy [2]. Different strategies to dilate the tracheal breach are utilized in the Percu Twist™technique (Rüsch, Kernen, Germany) and in the Griggs method (Portex^® ^Smiths Medical International Ltd., Hythe, Kent, UK) [5,10-12]. In the Percu Twist™technique a tracheal stoma is created by a screwlike dilating device, whereas in the method introduced by Griggs a pair of forceps are used to dilate the tracheal breach [5,9-14]. Compression of the anterior tracheal wall is minimal in both methods potentially reducing injury to the posterior wall [12,13].

The aim of this study is to describe a technical modification of percutaneous tracheostomy that combines the principles of the Percu Twist™ and the Griggs-Portex^® ^methods.

## Materials and methods

This prospective case series study was approved by the Research Ethics Committee of the Universidade Federal de Minas Gerais, Belo Horizonte, Brazil (resolution number: ETIC 0392.0.203.000-10), in accordance with the WMA Declaration of Helsinki - Ethical Principles for Medical Research Involving Human Subjects. A family member responsible for the patient was contacted for informed consent prior to the procedure. Data were obtained prospectively (data collecting sheet) at the Risoleta Tolentino Neves University Hospital Trauma Center (affiliated to the Universidade Federal de Minas Gerais) from June 1, 2010 to March 31, 2011. Inclusion criteria were age 18 years or older and an indication for tracheostomy. All percutaneous tracheostomies, as well as, ultrasound and bronchoscopy were performed by the authors JBRN, AJO, MPN. General surgery residents of the Federal University of Minas Gerais performed the procedure under supervision. Data included demographics, indication for tracheostomy, body mass index (BMI), thyromental distance (measured from the thyroid notch to the inferior border of the mentum), tracheostomy tube size (internal diameter), and acute complications. Procedure time was recorded by a nurse with a digital stopwatch. It is our practice to correct the coagulation parameters of the patients prior to percutaneous tracheostomy. Therefore, we also reviewed the data pertaining to prothrombin time, activated partial prothrombin time, platelet count, and the international normalized ratio (INR).

### Modified Percutaneous Tracheostomy Technique

**Figure 1:**
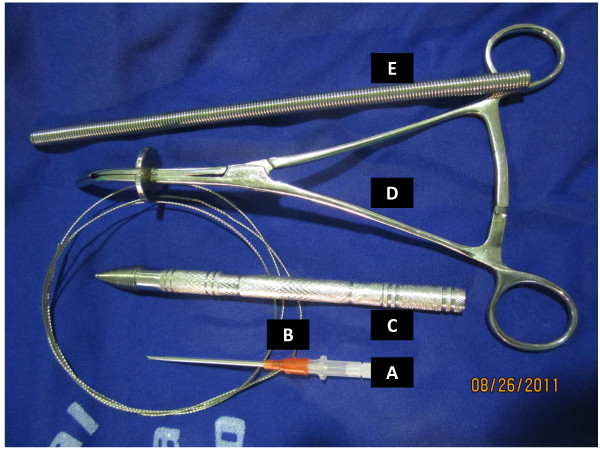
**The instruments used for percutaneous tracheostomy**. (A) The 14G intravenous catheter- Jelco^®^; (B) the guidewire; (C) the threaded tip dilator; (D) the self retaining retractor; (E) the spherical tip flexible introducer.

The instruments used for percutaneous tracheostomy in this work were manufactured from stainless steel and are reusable (Figure 1). All mechanically ventilated patients are sedated (midazolan 1-2 mg and fentanyl 100-200 mcg), paralyzed (pancuronium 0.04-0.1 mg/Kg), and placed on 100% oxygen starting 5 minutes before and until 5 minutes after the completion of the procedure. Positive end expiratory pressure (PEEP) setting is not changed for the procedure. A pulse oximeter (Datex-Ohmeda Inc., Tewksbury, MA) is used to assess hemoglobin oxygen saturation. Trauma patients with cervical spine cleared by physical examination in addition to radiograph of the neck and/or computed tomography scan, have their neck slightly extended by a 10 cm high pillow placed underneath the shoulders. Otherwise, the patient's neck and the bed are maintained in neutral position. If the cervical collar has to be removed a head immobilization device is used to stabilize the cervical spine (HeadBed™ II, Laerdal do Brasil, Barueri, SP, Brazil).

**Figure 2:**
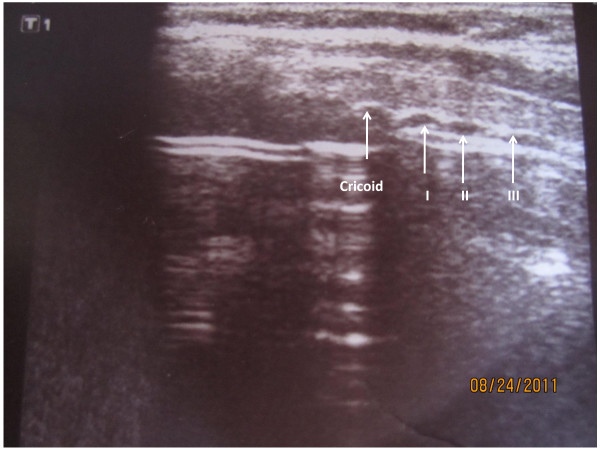
**Ultrasound image of the trachea**. Longitudinal view of the trachea shows the cricoid cartilage and the tracheal rings; important anatomical references to localize the site of puncture of the trachea. The endotracheal tube has been pulled back. I, II, III (first, second, and third tracheal rings); image obtained with an 8 MHz vascular probe.

**Figure 3:**
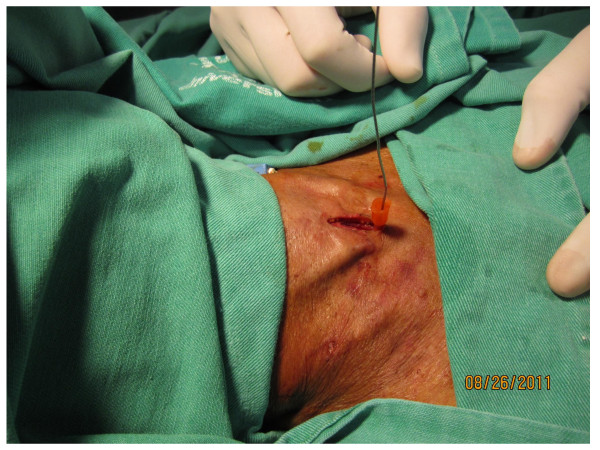
**The guidewire in position**. The guidewire is passed into the tracheal lumen through the 14 G intravenous catheter.

**Figure 4:**
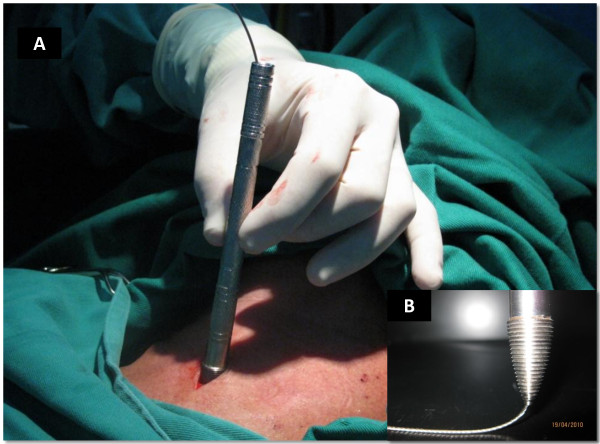
**The threaded tip dilator**. Picture (A) shows the threaded tip dilator in the incision. Insert (B) depicts how the guidewire, passing through the tip of the threaded dilator, prevents the threads from "catching" other structures.

**Figure 5:**
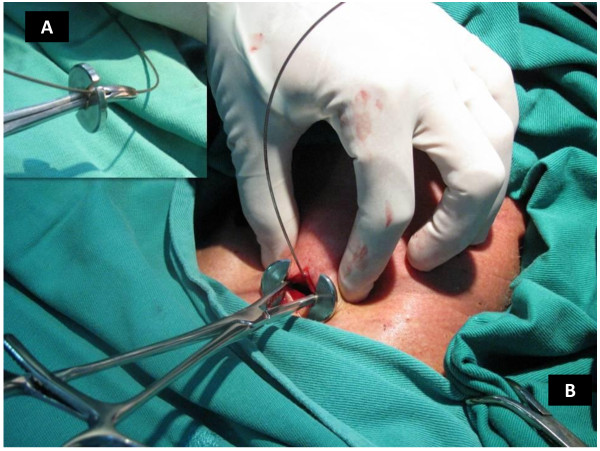
**The self-retaining retractor**. Insert (A) depicts how the self-retaining retractor is passed over the guidewire in locked position. Picture (B) shows how the retractor enables hands free lateral retraction of the pre-tracheal soft tissue, and the aperture on the anterior tracheal wall. The limiter ridge prevents insertion of the retractor too far into the trachea.

**Figure 6:**
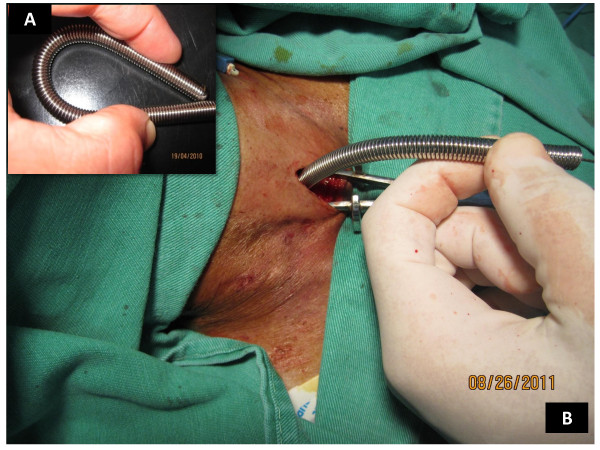
**The spherical tip flexible introducer**. Insert (A) depicts the elastic property of the introducer constructed with a circular helical spring. Picture (B) shows the flexible introducer positioned in the trachea facilitated by the self-retaining retractor.

**Figure 7:**
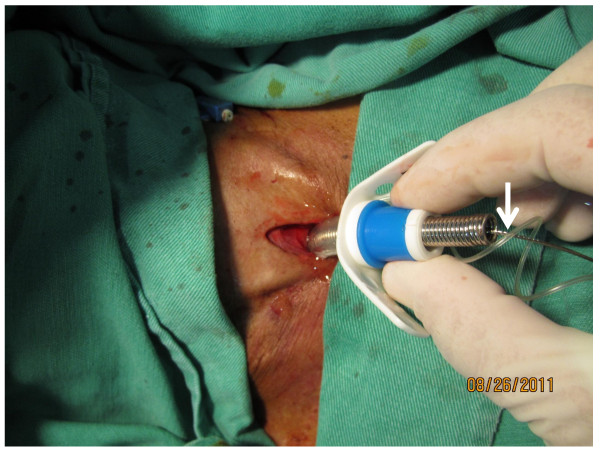
**Insertion of the tracheostomy tube in the trachea**. Picture shows the insertion of the tracheostomy tube in the trachea over the spherical tip flexible introducer. Arrow depicts the guidewire inside the introducer.

The operative site is prepared with 10% povidone iodine solution and infiltrated with 2% lidocaine (Astra Zeneca, Sao Paulo, Brazil);10 mg/Kg maximum dose. Ultrasound of the neck is performed with an 8 MHz Ultrasound Vascular Probe (Toshiba Nemio XG, Toshiba America Medical Systems, Inc. Tustin, CA) to assess vascular anatomy of the anterior neck, the distance between the skin and the trachea in the midline, as well as, to identify the thyroid isthmus, the cricoid cartilage, and 1^st ^to 3^rd ^tracheal rings (Figure 2). The endotracheal tube is pulled back under ultrasound guidance until the cuff is at the level of the thyroid cartilage, thus avoiding puncture of the cuff. The cricoid cartilage is palpated, and a 1.5 cm vertical incision is made immediately below that point. The subcutaneous tissue is bluntly dissected with hemostats until the pretracheal fascia is exposed. The trachea is punctured between the first and second, or the second and the third tracheal rings with a 14 G intravenous catheter needle (Jelco^®^; Medex, Carlsbad, CA) attached to a fluid filled 10 cc syringe, under ultrasound guidance. As soon as aspiration of air into the syringe is confirmed, the intravenous catheter is advanced into the trachea and the needle is removed. A flexible guidewire is gently passed through the intravenous catheter into the trachea; the catheter is removed afterwards (Figure 3). Ultrasound is once again used to verify endotracheal positioning of the guidewire. A threaded dilator (6 mm internal diameter) is advanced into the trachea, over the guidewire, for approximately 1 cm by clockwise rotation; minimal pressure is exerted on the anterior tracheal wall (Figure 4). The threaded dilator is removed by counter clockwise rotation after air escape through the lumen is detected. A self-retaining retractor forceps, with a limiter ridge, is passed over the guidewire into the trachea in locked position. The retractor is opened to enlarge the tracheal breach laterally, and to maintain the tracheal orifice open (Figure 5). A flexible, spherical tip introducer (6 mm internal diameter) is inserted into the airway under direct vision, facilitated by the retractor which is removed afterwards (Figure 6). A tracheostomy tube is placed inside the trachea passing over the guidewire and the flexible introducer (Figure 7). At this point the flexible introducer and the guidewire are removed, the cuff is inflated, and the patient is ventilated via the tracheostomy tube. The endotracheal tube is completely removed after adequate ventilation is confirmed by end-expiratory volume on the ventilator and auscultation of the patient. The tracheostomy cannula is secured in place with a neckband, and a chest radiograph is performed. Statistical analysis was performed using Graph Pad Prism (GraphPad Prism Software, Inc., San Diego, CA). Data are reported as the mean ± SEM and percentages.

## Results

During the study period, 100 patients underwent percutaneous tracheostomy by the modified technique described in this study. All percutaneous tracheostomies were performed on intubated patients at the bedside. Ninety patients (90%) underwent the procedure in the ICU. The remaining 10 patients were in another hospital location: 4 patients were in the hospital step-down unit, 3 in the trauma room, and 3 in the post-anesthesia recovery room. Demographic data showed that the majority of the patients were men (68%) with a mean age of 49 ± 2.2 years. The mean BMI of the patients was 25.6 ± 2.1, and the thyromental distance was 6.2 ± 0.3 cm. The pretracheal tissue thickness was 1.5 ± 0.7 cm. Twenty five percent of the percutaneous tracheostomies were performed on trauma patients, and18% on acute care surgery non-trauma patients. The remaining patients were admitted to the hospital because of clinical (29%) or neurologic (28%) related diseases. The most common indication for percutaneous tracheostomy (95 patients) was the need for prolonged ventilatory support, with a mean intubation period of 9.5 ± 4.2 days. Five patients underwent the procedure because of severe maxillofacial trauma. Percutaneous tracheostomy procedure time was 5.1 ± 0.3 minutes, assessed from the time of skin incision to the time of placement of the tracheostomy tube inside the airway. A tracheostomy tube size 9.0 mm (internal diameter) was used in 70 patients (70%), a size 8.5 mm (internal diameter) was used in 20 patients, and a tube size 8.0 mm in the remaining patients.

The mean prothrombin time prior to the procedure was 80.9 ± 5.5% (Quick Value), the activated partial thromboplastin time was 30.6 ± 1.9 seconds, the mean INR was 1.2 ± 0.1, and platelet count was 216.3 ± 35.5 x10^3^/uL.

Patients were followed for an average of 6.6 ± 2.2 days for complications. There were no inadvertent extubations, tracheostomy cannula misplacements and false passages, or oxygen desaturation below 95%, in the patients of the study. Furthermore, post-procedure chest radiograph showed no pneumothorax and no subcutaneous emphysema in the neck. There were two bleeding complications (2%) that resolved with dressing changes. Hemodialysis and anticoagulation shortly after the procedure could have contributed to the bleeding episode in one of the cases. There were no conversions to open surgical tracheostomy, and no deaths related to percutaneous tracheostomy in this study.

Bronchoscopy was performed in the first ten patients. In all cases, midline tracheal puncture, proper positioning of the thread tip dilator, as well as, integrity of the posterior wall of the tracheal were confirmed during the procedure.

## Discussion

Percutaneous tracheostomy via the modified Seldinger technique was first described in 1969, and has gained several variants since then [2,5-17]. One of the main advantages of percutaneous tracheostomy is bedside performance, thus eliminating the expenses and logistics involved in operating room set-up usually required for open surgical tracheostomies. Furthermore, several investigators have reported shorter procedure times and lower complication rates with percutaneous tracheostomy compared to open surgical tracheostomy [4,11,14,15,18-22].

The percutaneous tracheostomy method described in this study combines technical principles common to other well consolidated techniques, particularly the Percu Twist™, and the Griggs-Portex^® ^procedures; and to a lesser extent the Schachner method [2,4,5,7,10,23-25].

Our experience of 100 cases underscores three important features of the technical variation described herein. First is the capability to produce the initial breach on the trachea smoothly, with minimal compression, facilitated by the fine threads on the dilator. Additionally, the anterior tracheal wall is pulled away from the posterior wall as the dilator is threaded into the trachea, thus reducing posterior wall injury. Furthermore, passage of the guidewire through the tip of the dilator prevents the threads from "catching" the posterior wall, also reducing inadvertent injury (Figure 4).

The second feature is the capability to maintain hands-free retraction of the pre-tracheal soft tissue, and the tracheal aperture, with the self retaining retractor. The device enables controlled lateral dilation of the tracheal breach up to 2 cm maximum, thereby preventing excessive dilatation. Interestingly, a safety evaluation study in adult cadavers demonstrated that the mean force required to dilate the trachea 1.5 to 2 cm with a Griggs forceps, was two times that for therapeutic tracheal dilatation and three times the force required for tracheal disruption (31.6 N vs. 97.7 N), respectively [26]. The strategic location of the limiter ridge on the retractor (1.5 cm from the tip) is an additional safety feature to prevent insertion of the retractor too far into the trachea, and posterior wall injury.

The third feature relates to the flexible introducer which is constructed with a circular helical spring that confers elasticity, and enough rigidity to safely avoid false passage and tracheostomy tube misplacement. Tracheostomy tubes of 7 to 9.5 mm internal diameter can be passed over the introducer and placed inside the airway.

Even though the percutaneous tracheostomy procedure described in this study incorporates technical principles of at least two different methods the mean procedure time (5.1 minutes) was consistent with single dilator techniques reported by others [10,13,21,27].

Acute complications with the percutaneous tracheostomy method described by us were restricted to hemorrhage. The post-procedure bleeding rate of 2% in our study is comparable to other reports (1.6 - 4%) [3-5,10,11,15,18,19,23,24]. Even though comparison of the method described herein was not the purpose of this study, a contemporary analysis of 30 open surgical tracheostomies performed in our institution showed a 4% incidence of post-procedure bleeding, 50% of those cases required a surgical intervention to control the hemorrhage (unpublished data- Joao B. Rezende-Neto). On the contrary, none of the percutaneous tracheostomy patients who had a bleeding complication required a surgical intervention in the present study. Interestingly, prothrombin (Quick Value) time and INR were equivalent among the patients, respectively; 80.9 ± 5.5% in percutaneous tracheostomy vs. 87.2 ± 3.1% in open surgical tracheostomy patients (*p *= 0.27, Student's *t*-Test), and 1.2 ± 0.1 in percutaneous tracheostomy vs. 1.3 ± 0.15 in open surgical tracheostomy patients (*p *= 0.64, Student's *t*-Test). Furthermore, time to perform time to perform percutaneous tracheostomy was significantly shorter than that of open surgical tracheostomy (5.1 ± 0.3 minutes vs. 12.2 ± 1.4 minutes; *p *< 0.001, Student's *t*-Test)

Several studies highlight the importance of bronchoscopy to reduce complications during percutaneous tracheostomies, and most institutions routinely perform the procedure under bronchoscopic guidance [4,11,18,19,24,28-32]. Unfortunately, our institution did not have bronchoscopy routinely available during the study period.

Even though bronchoscopy is considered an important adjunct to percutaneous tracheostomy, that enables confirmation of midline puncture of the trachea, correct position of the guidewire and the tracheostomy tube, as well as, visualization of posterior tracheal wall injury, it is not without complications [4,31,33,34]. Studies have shown that bronchoscopy can cause hypoventilation that leads hypercarbia and respiratory acidosis during percutaneous tracheostomy [12,35,36]. Nonetheless, percutaneous tracheostomy without bronchoscopic guidance remains a controversial issue [4,12,19,29,31,34,37-40].

Investigators who forgo bronchoscopy during percutaneous tracheostomy usually implement additional measures to compensate for the lack of intraluminal visualization of the trachea [12,18,37-39,41-44]. For example, dissection of the subcutaneous tissue down to the pre-tracheal fascia prior to tracheal puncture, palpation of the trachea through the incision during endotracheal tube positioning and tracheal puncture, verification of free mobility of the guidewire throughout the procedure, and capnography assessed at the puncture site [12,18,37-39,41-44]. Additionally, ultrasound has become an increasingly used adjunct to percutaneous tracheostomy when bronchoscopy is not available, particularly in obese patients. Several studies have shown that sonography is helpful to delineate the anatomy of the neck prior to the procedure; particularly the thyroid gland, pre-tracheal vascular structures, the thyroid and cricoid cartilages, and the first three tracheal rings [18,24,45-48]. Real-time ultrasound guidance makes it possible to follow the needle path during tracheal puncture, and the final position of the tracheostomy tube [46,49-51]. Because of unavailability of bronchoscopy in our institution, real time ultrasound was the main adjunct to the percutaneous tracheostomy technique described in this study.

There are several limitations to this study. There is the possibility that the low complication rate with our technique could be linked to the favorable anatomic features of our patients, defined by a mean thyromental distance > 6 cm and a mean BMI of 25.6. Previous studies have shown that a short thyromental distance and a high BMI are useful predictors of difficult intubation and a challenging surgical airway [52-55]. Another point is the coagulation parameters of our patients. There is the possibility that the low incidence of bleeding complications with the technique would not have been obtained if patients with abnormal coagulation parameters were included in the study. Unfortunately we did not assess the patients for other risk factors, such as, pre-procedure positive end expiratory pressure > 10 cm H_2_O or fraction of inspired oxygen > 50% [4]. Even though, the follow-up period in the study was sufficiently long for the determination of acute complications, it did not extend long enough for detection of long term complications, such as post-procedure tracheal stricture, associated with our method. That limitation is corroborated by previous reports that show late symptoms related to percutaneous tracheostomies in up to 20% of the patients followed for 39 months [4,20,46,56]. Furthermore, only 10 patients in our study underwent bronchoscopic guided percutaneous tracheostomy, thus significantly limiting our capability to determine complications and the shortcomings of the technique. Even though the technique can be performed without bronchoscopic guidance, it should be used whenever available, particularly during the learning curve which is of approximately 20 patients for percutaneous dilatational tracheostomy [57]. Lastly, we did not compare our method to other percutaneous tracheostomy techniques; unfortunately, we do not have commercial tracheostomy kits readily available at our institution. Nonetheless, our results were in accordance with the data from other publications.

## Conclusions

In our experience, percutaneous tracheostomy performed with the technical modification described in this study, is safe and simple to execute. However, long term follow-up for complications, is warranted. Additionally, reproducibility of results and a comparison to commercially available tracheostomy kits are required to further validate the method.

## Abbreviations

ICU: Intensive Care Unit; BMI: Body Mass Index; and INR: International Normalized Ratio.

## Competing interests

The Universidade Federal de Minas Gerais (Dr. Joao B. Rezende-Neto) filed a patent application for the technique and the device described in this manuscript (Patent Pending Number 902833073 - INPI - Brazil). All other authors declare that they have no competing interests in relation to this manuscript.

## Authors' contributions

Conception of the technique, design of the study, acquisition and interpretation of data, and drafting of the manuscript (JBRN); acquisition and interpretation of data, and execution of bedside ultra-sound (AJO); acquisition and interpretation of data, and drafting of the manuscript (MPN); interpretation of data and drafting of the manuscript (FAB); drafting of the manuscript and revision for important intellectual content (SBR). All authors read and approved the final manuscript.

## Authors' information

JBRN - Associate Professor Department of Surgery Universidade Federal de Minas Gerais, Brazil. Chief of Trauma and Acute Care Surgery Risoleta Tolentino Neves Hospital.

AJO - Intensivist Risoleta Tolentino Neves Hospital.

MPN - Trauma Surgeon Risoleta Tolentino Neves Hospital.

FAB - Assistant Professor of Internal Medicine Universidade Federal de Minas Gerais, Brazil. Chief of Critical Care Medicine Risoleta Tolentino Neves Hospital.

SBR - Associate Professor of Surgery and Critical Care Medicine University of Toronto and Sunnybrook Hospital, De Souza Trauma Research Chair.
